# Increased Expression and Protein Divergence in Duplicate Genes Is Associated with Morphological Diversification

**DOI:** 10.1371/journal.pgen.1000781

**Published:** 2009-12-24

**Authors:** Kousuke Hanada, Takashi Kuromori, Fumiyoshi Myouga, Tetsuro Toyoda, Kazuo Shinozaki

**Affiliations:** 1 Gene Discovery Research Group, RIKEN Plant Science Center, Yokohama, Kanagawa, Japan; 2 Bioinformatics and Systems Engineering Division, RIKEN, Yokohama, Kanagawa, Japan; University of Arizona, United States of America

## Abstract

The differentiation of both gene expression and protein function is thought to be important as a mechanism of the functionalization of duplicate genes. However, it has not been addressed whether expression or protein divergence of duplicate genes is greater in those genes that have undergone functionalization compared with those that have not. We examined a total of 492 paralogous gene pairs associated with morphological diversification in a plant model organism (*Arabidopsis thaliana*). Classifying these paralogous gene pairs into high, low, and no morphological diversification groups, based on knock-out data, we found that the divergence rate of both gene expression and protein sequences were significantly higher in either high or low morphological diversification groups compared with those in the no morphological diversification group. These results strongly suggest that the divergence of both expression and protein sequence are important sources for morphological diversification of duplicate genes. Although both mechanisms are not mutually exclusive, our analysis suggested that changes of expression pattern play the minor role (33%–41%) and that changes of protein sequence play the major role (59%–67%) in morphological diversification. Finally, we examined to what extent duplicate genes are associated with expression or protein divergence exerting morphological diversification at the whole-genome level. Interestingly, duplicate genes randomly chosen from *A. thaliana* had not experienced expression or protein divergence that resulted in morphological diversification. These results indicate that most duplicate genes have experienced minor functionalization.

## Author Summary

The relationship between morphological and molecular evolution is a central issue to the understanding of eukaryote evolution. In particular, there is much interest in how duplicate genes have contributed to morphological diversification during evolution. As a mechanism of functionalization of duplicate genes, differentiation of both gene expression and protein function are believed to be important. Although it has been reported that both expression and protein divergence tend to increase as a duplication ages, it is unclear whether expression or protein divergence in duplicate genes is greater in those genes that have undergone functionalization compared with those that have not. Here, we studied 492 duplicate gene pairs associated with various degrees of morphological diversification in *Arabidopsis thaliana*. Using these data, we found that the divergence of both expression and protein sequence were important sources for morphological diversification of duplicate genes. Although both mechanisms are not mutually exclusive, our analysis suggested that expression divergence is the minor contributor and protein divergence is the major contributor to morphological diversification. However, the expression or protein sequence of randomly chosen duplicate genes did not show significant divergence that resulted in morphological diversification. These results indicate that most duplicate genes experienced minor functionalization in the genome.

## Introduction

Duplicate genes rarely exhibit *de novo* functions (neofunctionalization); more usually, the functions of the original gene are split into multiple functions among the duplicate genes (subfunctionalization) [1]–[5]. Such functionalization through gene duplication is considered to be an important source of diversification in complex organisms [6]. As a mechanism of functionalization in duplicate genes, differentiation of both gene expression and protein function are thought to be important. In particular, differential patterns of gene expression among paralogs are widely believed to play a prominent role in morphological diversification, because such differences are essential for development [7]–[10]. However, substantial amounts of data support morphological diversification through divergence of protein function [11].

Many researchers have studied divergence of either expression or protein function in duplicate genes at the genome scale [12]–[24]. Although divergence of either expression or protein sequence tends to increase as a duplication ages, it is unclear whether either expression or protein divergence in duplicate genes has been elevated by functionalization. Therefore, it is of interest to compare the divergence rate of either expression pattern or protein sequence of duplicate genes of the same age that have and have not undergone functionalization. If divergence of both expression and protein function are important sources for functionalization, the divergence rate of both should be higher in duplicate genes that have undergone functionalization compared with those that have not.

*A. thaliana* is an excellent model organism for addressing the above issue because it has a highly duplicated genome and many knock-out mutants have been generated. Here, to address how duplicate genes have contributed to morphological evolution, we classified *Arabidopsis* duplicate genes into high, low and no morphological diversification groups based on knock-out data, and examined the divergence rates of both expression pattern and protein sequence among the three morphological diversification groups.

## Results/Discussion

### Identification of paralogous gene pairs associated with morphological diversification

**Figure 1:**
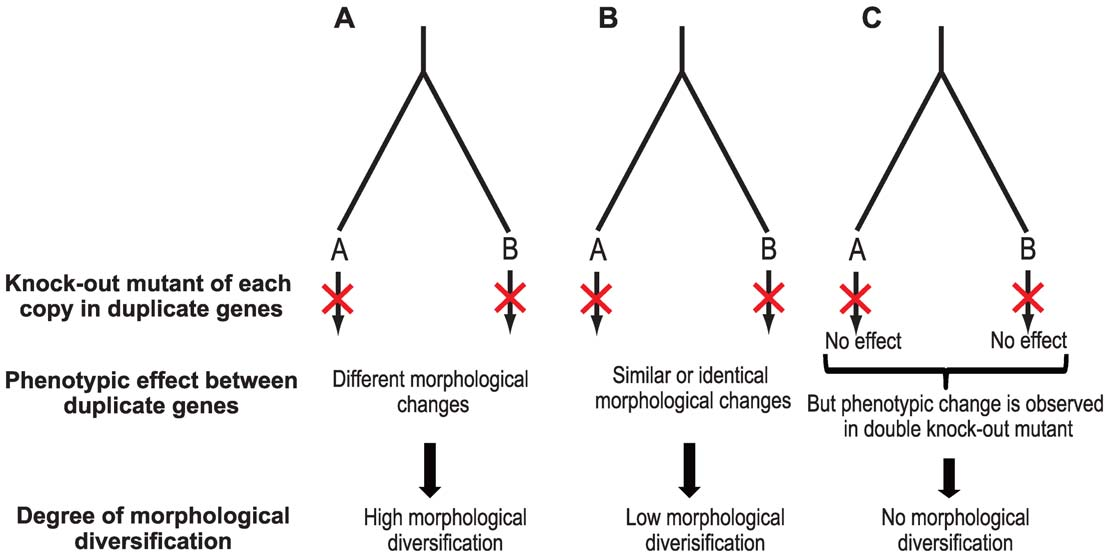
Paralogous gene pairs with high, low, and no morphological diversification. (A) Paralogous gene pairs with different knock-out phenotypes are defined to have high morphological diversification. (B) Paralogous gene pairs with similar or identical knock-out phenotypes are defined to have low morphological diversification. (C) Paralogous gene pairs in which morphological changes are observed only upon the deletion of multiple paralogous genes but not by the deletion of each gene individually are defined to have no morphological diversification.

From the literature and from our earlier work (see Materials and Methods) [25],[26] we identified 398 pairs of duplicate genes in which the knock-out mutant of either gene in a pair induced abnormal morphological changes relative to wild type. Abnormal morphological changes were classified into seed, vegetative and reproductive phenotypes on the basis of the definition of Meinke et al [27]. When the knock-out phenotype is totally different between genes in a paralogous gene pair, it is reasonable to assume that functionalization occurred after gene duplication (Figure 1A). For example, the knock-out mutant of AT4G09820 and AT5G41315 genes induced a yellow seed coat in the reproductive stage and a reduction of trichomes in the vegetative stage, respectively. Therefore, the knock-out phenotype is completely different between AT4G09820 and AT5G41315 because two abnormal phenotypes appeared in different developmental stages. Thus, paralogous genes with different phenotypes (morphological differences between phenotypes) are defined to have high morphological diversification. It is more common, however, to observe knock-out phenotypes that are similar or identical between paralogous genes (Figure 1B). For example, the knock-out mutants of AT1G62830 and AT3G10390 genes both induced late flowering. Although the knock-out phenotype of the two genes is similar, there would appear to be functionalization in such paralogous genes because a morphological change resulting from the deletion of one gene occurs when there is no or little functional redundancy between the paralogous genes. We, therefore, thought that such paralogous genes had some degree of functionalization after gene duplication. However, it is likely that similar or identical phenotypes indicate paralogous genes that have lower functionalization compared with paralogous genes with different phenotypes. Therefore, paralogous genes with either similar or identical phenotypes (morphological changes within phenotypes) were defined to have low morphological diversification. In this study, we identified 163 and 235 paralogous gene pairs associated with high and low morphological diversification, respectively. As a control set, we focused on paralogous gene pairs in which abnormal morphological changes are observed only upon the deletion of multiple paralogous genes but deletion of each gene separately did not induce abnormal morphological changes (Figure 1C). For example, the double knock-out mutant of AT3G58780 and AT2G42830 exhibits fruit dehiscence but knock-out of each gene alone did not induce abnormal morphological changes. Such paralogous gene pairs are likely to have some degree of functional redundancy. We, therefore, defined these paralogous gene pairs as having no morphological diversification. The number of paralogous gene pairs identified without morphological diversification was 94. Thus, we identified a total of 492 paralogous gene pairs associated with the three kinds of morphological diversification (Table S1).

### Divergence of gene expression in paralogous gene pairs associated with morphological diversification

**Figure 2:**
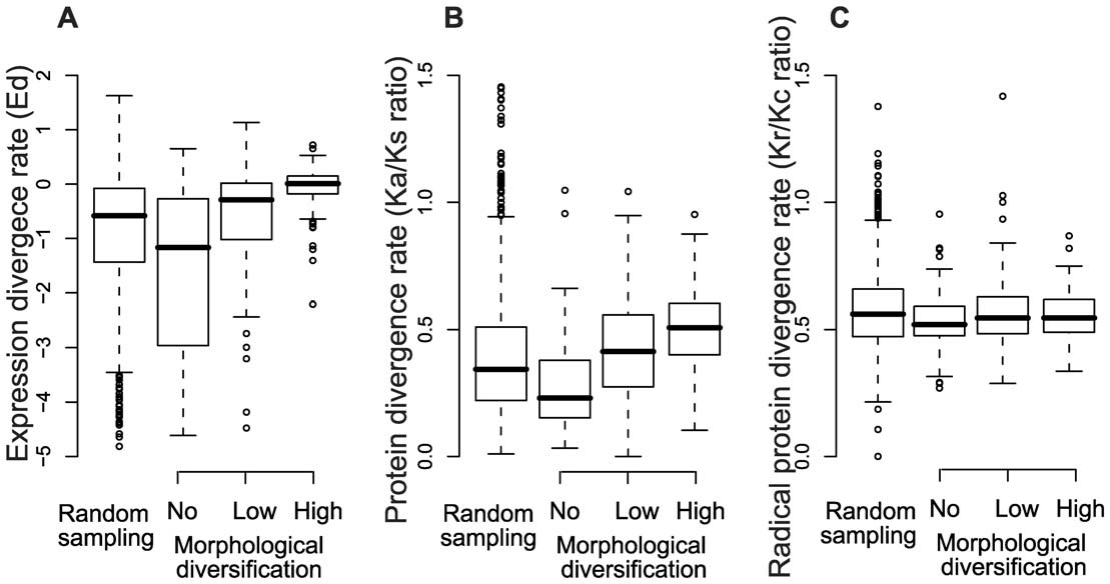
Divergence rate of expression and protein sequence in paralogous gene pairs. (A) Relationship between expression divergence (*Ed*) and morphological diversification (defined in the main text). *Ed* is log ((1−R)/(1+*R*))/Ks, where *R* is the correlation coefficient of paralogous gene pairs among different experimental conditions and Ks is synonymous distance. (B) Relationship between ratio of Ka (nonsynonymous distance) to Ks in paralogous gene pairs and morphological diversification. (C) Relationship between ratio of Kr (radical nonsynonymous distance) to Kc (conservative nonsynonymous distance) and morphological diversification. The random sample included 1,000 pairs of paralogs. The distributions of Ed, Ka/Ks ratio and Kr/Kc ratio are shown as box plots with the solid horizontal line indicating the median value, the box representing the inter quartile range (25%–75%), and the dotted line indicating the first to the 99th percentile.

To examine the expression pattern divergence for a paralogous gene pair, we obtained intensities of gene expression by microarray analysis under 634 conditions. Expression divergence in a pair of genes is usually inferred by 1 minus R (Pearson's coefficient of correlation) of the expression intensities among experimental conditions. Here, we transformed the value as log((1−*R*)/(1+*R*)), because the transformation is more sensitive for examining expression differences [19]. When we applied the log((1−*R*)/(1+*R*)) values to paralogous gene pairs among the three morphological diversification groups, the log((1−R)/(1+R)) values increased as morphological diversification increased (Figure S1). However, the relationship may be strongly influenced by duplication age (sequence divergence) in the case that morphological diversification increases as sequence divergence increases. We, therefore, investigated sequence divergence in paralogous gene pairs by examining synonymous (Ks) and nonsynonymous (Ka) distance among morphological diversification groups [28]. Consequently, both synonymous and nonsynonymous distances increased as morphological diversification increased (P<0.01 by Wilcoxon's test; Figure S1 and Table S2). To minimize the effect of duplication age, log((1−*R*)/(1+*R*)) was divided by Ks. This is because expression divergence is expected to increase as duplication timing becomes earlier and Ks increases in a nearly linear fashion with duplication age [17],[19],[24]. *Ed* (log ((1−*R*)/(1+*R*))/Ks) is an indicator of the expression divergence rate between a paralogous gene pair: high and low *Ed* indicates high and low expression divergence at the same duplication age, respectively. When we calculated *Ed* between a paralogous gene pair in the three morphological diversification groups, *Ed* increased as morphological diversification increased (Figure 2A). *Ed* differed significantly between each pair of morphological diversification groups (*P*<0.01 by Wilcoxon's test; Table S2), suggesting that expression divergence is an important source for morphological diversification of duplicate genes.

There are genetic and epigenetic factors that are the source of expression divergence. Since the differentiation of cis-regulatory elements can be a major genetic effect, we examined the proportion of known cis-regulatory elements that overlap in the promoter regions of paralogous gene pairs [29]. The proportion of cis-regulatory elements that overlap decreased as morphological diversification increased (Figure S2). The proportion of overlapping cis-regulatory elements differed significantly between each pair of morphological diversification groups (*P*<0.05 by Wilcoxon's test; Table S2 and Figure S2), indicating that the divergence of cis-regulatory elements contributes to morphological diversification. With respect to epigenetic factors, we investigated the proportion of methylated cytosines to non-methylated cytosines in the promoter regions of paralogous genes [30]. The proportional difference in paralogous gene pairs did not significantly differ between each pair of morphological diversification groups (Table S2 and Figure S2), indicating that an epigenetic effect through methylation is unlikely to contribute to morphological diversification. Taken together, expression divergence led by the differentiation of cis-regulatory elements is an important source for morphological diversification in duplicate genes.

### Protein divergence in paralogous gene pairs associated with morphological diversification

Because duplication age (sequence divergence) between paralogous gene pairs increased as morphological diversification increased (Figure S1), we examined divergence rates of protein sequences of the same duplication age. Divergence rates of protein sequences are commonly inferred from selection pressure in coding sequences, i.e. the ratio of the non-synonymous substitution rate (Ka) to Ks. High and low Ka/Ks ratios indicate high and low protein divergence rates at the same duplication age, respectively [28]. When we applied the Ka/Ks ratio to paralogous gene pairs within the three morphological diversification groups, the Ka/Ks ratio increased as the morphological diversification increased (Figure 2B). The Ka/Ks ratio differed significantly between each pair of morphological diversification groups (*P*<0.01 by Wilcoxon's test; Table S2), suggesting that protein divergence is an important source for morphological diversification of duplicate genes.

To analyze the kinds of amino acid replacements that have occurred during morphological diversification, we classified all amino acid replacements as either ‘chemical radical’ or ‘conservative’ on the basis of an amino acid classification generated in an earlier report [31]. We examined the ratio of the radical nonsynonymous substitution rate (Kr) to the conservative nonsynonymous substitution rate (Kc). Interestingly, the Kr/Kc ratios of all types of paralogous gene pairs were similar (Figure 2C and Table S2), indicating that paralogous gene pairs with either high, low or no morphological diversification tend to have the same level of radical protein divergence. The Kr/Kc ratio based on this amino acid classification is significantly correlated with the Ka/Ks ratio at the whole genome level [31]. Therefore, radical changes become restricted in paralogous gene pairs with higher morphological diversification. One explanation for this restriction is that radical changes do not affect morphological diversification. However, some reports have shown that radical changes significantly influence functional divergence [23],[32]. Therefore, it does not seem to be a reasonable explanation. Another explanation is that radical changes may induce serious functional errors. To maintain duplicate genes that encode functional proteins, radical changes may be too deleterious. Therefore, paralogous gene pairs involved in higher morphological diversification may be subject to purifying selection against radical amino acid changes.

### Divergence rate of expression pattern versus protein sequence in paralogous gene pairs associated with morphological diversification

To compare the divergence rate of expression pattern with that of protein sequence in paralogous gene pairs associated with morphological diversification, we focused on paralogous gene pairs without morphological diversification because the divergence rate of expression pattern and/or protein sequence in these duplicate genes has little effect on morphological diversification. Therefore, the top 5% of *Ed* and Ka/Ks ratios for paralogous gene pairs without morphological diversification were defined to be the threshold of higher divergence rate of expression pattern and protein sequences, respectively. We then counted the numbers of paralogous gene pairs with a higher divergence rate in each of the high and low morphological diversification groups (Table 1). To make the relative roles clear, we simply compared the observed ratio between paralogous gene pairs with only higher expression divergence and those with only higher protein divergence, assuming no bias between expression and protein divergence in either high or low morphological diversification groups. Interestingly, the number of paralogous gene pairs (37 in either high or low morphological diversification groups) with a protein divergence but no expression divergence was significantly higher than the number of paralogous gene pairs (62 in either high or low morphological diversification groups) with a higher expression divergence but no protein divergence, as determined by the chi-square test (P<0.05). These results indicate that paralogous gene pairs with a higher divergence rate of protein sequence contribute to morphological diversification more effectively than those with a higher divergence rate of expression. The inference from these results is that protein sequence plays the major role (59–67%) and expression plays the minor role (33–41%) in morphological diversification.

**Table 1:** Number of paralogous gene pairs with a high divergence rate of protein sequence and/or expression in the high and low morphological diversification groups.

Morphological divergence	Protein	Divergent expression	Not divergent expression	*p*-value^c^
High	Divergent	16	30 (59%)^b^	0.21
	Not divergent	21 (41%)^a^	76	
Low	Divergent	13	32 (67%)^b^	0.02
	Not divergent	16 (33%)^a^	116	
High or Low	Divergent	29	62 (63%)^b^	0.01
	Not divergent	37 (37%)^a^	193	

**a** Proportion of paralogous gene pairs with a higher expression divergence but no protein divergence.

**b** Proportion of paralogous gene pairs with a higher protein divergence but no expression divergence.

**c** Null hypothesis is that the proportion of paralogous gene pairs with a higher expression divergence is the same proportion of paralogous gene pairs with a higher protein divergence.

We performed the same analysis using the top 10% of *Ed* and Ka/Ks ratios of paralogous gene pairs without morphological diversification as the threshold of higher divergence rate of expression pattern and protein sequences, and obtained essentially the same results (Table S3). Therefore, we believed that the relative rates of expression and protein divergence are stringent in morphological diversification.

### Divergence rate of expression and protein sequence in duplicate genes at the whole genome level

Finally, we addressed to what extent duplicate genes were associated with expression or protein divergence exerting morphological diversification at the whole genome level. To examine this question, we randomly chose 1000 pairs of paralogous gene pairs. We then compared *Ed* and Ka/Ks ratios among the 1000 random paralogous gene pairs and among paralogous gene pairs with high, low or no morphological diversification (Figure 2). Both *Ed* and Ka/Ks ratios for the random paralogous gene pairs were significantly lower compared with that for the paralogous gene pairs with high or low morphological diversification but were significantly higher compared with that for the paralogous gene pairs without morphological diversification (P<0.01 by Wilcoxon's test, (Figure 2A and 2B and Table S2). However, the Kr/Kc ratio was not different between any pair in the four categories (*P*>0.05 by Wilcoxon's test, Figure 2C and Table S2). As discussed earlier, the Kr/Kc ratio is not an indicator for functionalization, therefore, no difference is reasonable. These results suggest that duplicate genes have not experienced divergence of expression or protein sequence exerting morphological diversification on a genome-wide scale. It is, therefore, likely that most duplicate genes have experienced only minor functionalization, at least in *A. thaliana*.

### Concluding remarks

To understand to what extent molecular changes in duplicate genes have contributed to morphological diversification in *A. thaliana*, we examined the divergence rate of either expression pattern or protein sequence in duplicate genes associated with morphological diversification and found that both divergences are important sources in morphological diversification. Although both mechanisms are not mutually exclusive, our analysis suggested that changes of protein sequence play the major role and changes of expression pattern play the minor role in morphological diversification. However, randomly chosen duplicate genes have not experienced divergence of expression or protein sequence exerting morphological diversification. These results indicate that most duplicate genes have experienced minor functionalization and only a few duplicate genes are likely to be crucial to morphological evolution.

## Materials and Methods

### Identification of paralogous gene pairs associated with three kinds of morphological diversification

We used data from the available literature and from our bank of previously generated T-DNA insertional mutants [25],[26], to identify 1203 duplicate genes whose knock-out induced abnormal morphological changes relative to wild type. The nucleotide sequences of *A. thaliana* (TAIR7) were obtained from TAIR (www.arabidopsis.org). Duplicate genes were defined as proteins that matched other proteins in a BLAST search with E<1×10^−4^ [33]. We then classified the 1203 duplicate genes into 786 gene families by the Markov clustering algorithm (http://micans.org/mcl/). In every pair of each family, we examined the amino acid identity and the coverage (percentage of alignable regions). We found 405 paralogous gene pairs with amino acid identity >0.3 and coverage >0.5. Since tandem duplicates have a higher chance of exhibiting similar expression due to leaky expression or conserved sequences by gene conversion than non-tandem duplicates [34]–[36], we removed tandem duplicates from the 405 paralogous gene pairs. As reported earlier [37], tandem duplicates were defined as genes in any gene pair, T1 and T2, that (1) belong to the same gene family, (2) are located within 100 kb of each other, and (3) are separated by at most 10 nonhomologous (not in the same gene family as T1 and T2) genes. In this definition, we identified 7 tandem paralogous gene pairs. After removing these tandem paralogous gene pairs, we used 398 non-tandem paralogous gene pairs in this study. Note that each knock-out mutant of paralogous genes induced abnormal phenotypic changes.

To examine the degree of morphological diversification between the genes of the paralogous gene pairs, we classified morphological changes into seed, vegetative and reproductive phenotypes, according to the definition of Meinke et al [27]; the changes were defined as high (morphological changes between phenotypes) and low (morphological changes within phenotypes) morphological diversification. Briefly, seed, reproductive and vegetative phenotypes show visible changes in development. We identified 163 paralogous gene pairs associated with high morphological diversification and 235 associated with low divergence (Table S1).

As a control set, we identified from the literature165 duplicate genes that did not show morphological diversification. Absence of morphological diversification was defined as the observation of morphological change only upon the deletion of multiple paralogs; deletion of each gene separately did not induce morphological change. After removing tandem paralogous gene pairs, we found 95 paralogous gene pairs with amino acid identity >0.3 and coverage >0.5 (Table S1).

### Expression analysis

We obtained Affymetrix ATH1 data from the AtGenExpress expression atlas at TAIR (http://www.arabidopsis.org/). We compiled 1280 microarray datasets under 634 conditions, consisting of 82 different developmental stages, 72 biotic treatments, 285 abiotic treatments, 11 nutrient treatments, 81 hormone treatments, 40 chemical treatments, 21 cell cycle stages and 42 different genotypes. The array intensities were processed with the Bioconductor (http://www.bioconductor.org) affy package in the R software environment (http://www.r-project.org). Specifically, the array intensities were adjusted to reduce background with the mas5 function, and the normalize quantiles function was used for between-array normalization. The background-corrected and background-normalized intensities were used for further analysis.

### Divergence of cis-regulatory elements and methylation in promoter regions

We obtained the mapping data of known cis-regulatory elements in 1 kb promoter regions of all *A. thaliana* genes at ATCOECIS (http://bioinformatics.psb.ugent.be/ATCOECIS/) [29]. To examine the divergence of cis-regulatory elements in each paralogous gene pair, we used the proportion of overlapping cis-regulatory elements (the number of overlapping cis-regulatory elements over the number of observed cis-regulatory elements). To examine divergence of methylation in paralogous gene pairs, we obtained the mapping data of bisulfite-treated DNA sequences in the TAIR7 genome at NCBI Gene Expression Omnibus (GSM276809) [30]. The bisulfate-treatment converts cytosine to uracil in unmethylated cytosine sites but does not affect cytosine in methylated cytosine sites. Since the methylation of each cytosine site was determined multiple times, a methylated cytosine site was defined when that site is more often methylated than not. We calculated the proportion of methylated cytosine sites (the number of methylated cytosine sites over the number of observed cytosine sites) in promoter regions (500 bp upstream from either start codon or transcriptional start site) of all *A. thaliana* genes because the methylation of 500 bp upstream regions is considered to be sensitive for gene expression [30]. The proportional difference of methylated cytosine sites in a paralogous gene pair was used to represent the methylation divergence in a paralogous gene pair.

### Inference of protein divergence rates

Nucleotide sequences of *A. thaliana* (TAIR7) were obtained from TAIR (www.arabidopsis.org). Pairwise alignment was performed with the program CLUSTALW to align coding regions [38]. Ks and Ka between paralogous genes were estimated by the modified Nei–Gojobori method [28]. The transition/transversion ratio was estimated for each paralogous gene pair, and the ratio was then used to estimate Ka and Ks. To infer the ratio of the radical non-synonymous substitution rate (Kr) to the conservative non-synonymous substitution rate (Kc), we classified amino acids according to Hanada et al. 2007 [31]. Radical and conservative changes were defined as amino acid replacements between and within groups, respectively. The ratio of Kr to Kc for each paralogous gene pair was estimated by the Zhang method [39].

### Generation of randomly chosen paralogous gene pairs

We randomly chose genes from the total set of annotated A. thaliana genes (TAIR7). For a chosen gene, similarity searches were conducted against all annotated *A. thaliana* genes using BLASTP [33]. We aligned the chosen gene and all homologous genes identified in the BLASTP search using CLUSTALW and estimated the amino acid similarity among them [38]. We calculated the amino acid identity and the coverage (percentage of alignable regions) between the chosen gene and the matched gene with the highest identity. If the paralogous gene pair had amino acid identity >0.3 and coverage >0.5, we added the pair to a random set. We repeated this procedure until we obtained 1000 paralogous gene pairs.

## Supporting Information

Figure S1 Expression divergence, synonymous, and nonsynonymous distances among random paralogous gene pairs and among paralogous gene pairs with no, low, and high morphological diversification. (A) Relationship between expression divergence and morphological diversification (defined in the main text). Expression divergence is log ((1−R)/(1+R)), where R is the correlation coefficient of paralogous gene pairs among different experimental conditions. (B) Relationship between Ks and morphological diversification. (C) Relationship between Ka and morphological diversification. The random sample included 1000 pairs of paralogs. The distributions of expression divergence, Ks and Ka are shown as box plots with the solid horizontal line indicating the median value, the box representing the inter quartile range (25%–75%), and the dotted line indicating the first to the 99th percentile.
(0.25 MB PDF)

Figure S2 Divergence of cis-regulatory element and methylation of promoter regions among paralogous gene pairs with no, low, and high morphological diversification. (A) Relationship between proportion of overlapped cis-regulatory elements and morphological diversification. The proportion of overlapped cis-regulatory elements is the number of overlapped cis-regulatory elements over the number of observed cis-regulatory elements in promotor regions of two paralogous genes. (B) Relationship between proportional difference of methylation and morphological diversification. The proportional diffrerence of methylation is the difference of proportion of methylated cytosine in promoter regions of two paralogous genes. These distributions are shown as box plots with the solid horizontal line indicating the median value, the box representing the inter quartile range (25%–75%), and the dotted line indicating the first to the 99th percentile.
(0.22 MB PDF)

Table S1 Paralogous gene pairs with no, low, and high morphological diversification.
(0.05 MB PDF)

Table S2 Statistical difference (P. values) in Figure 2, Figure S1, and Figure S2.
(0.01 MB PDF)

Table S3 Number of paralogous gene pairs with a high divergence rate of protein sequence (more than the top 10% of Ed of paralogous gene pairs without morphological diversification) and/or expression (more than the top 10% of Ka/Ks ratios of paralogous gene pairs without morphological diversification) in the high and low morphological diversification groups.
(0.03 MB PDF)
