# Supplementary figures and images for: Increased Expression and Protein Divergence in Duplicate Genes Is Associated with Morphological Diversification
Source: PLoS Genet. 2009 Dec 24;5(12):e1000781. doi: 10.1371/journal.pgen.1000781 (PMC2788128; doi:10.1371/journal.pgen.1000781)

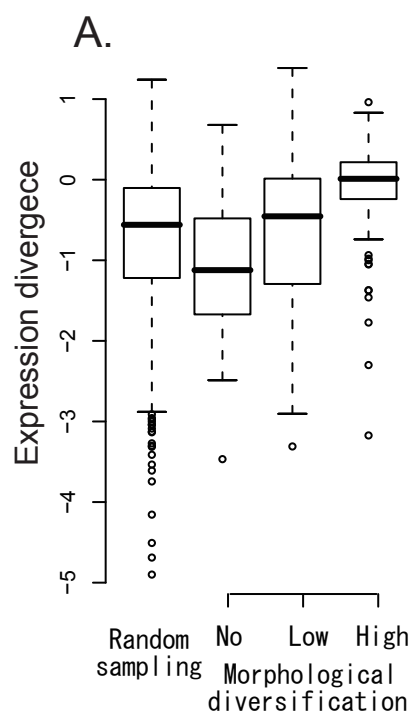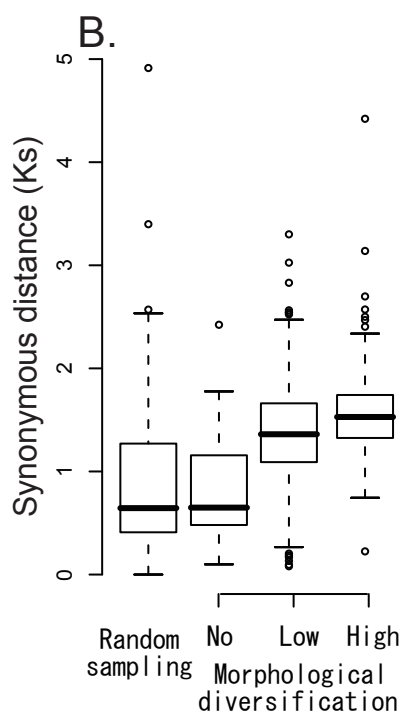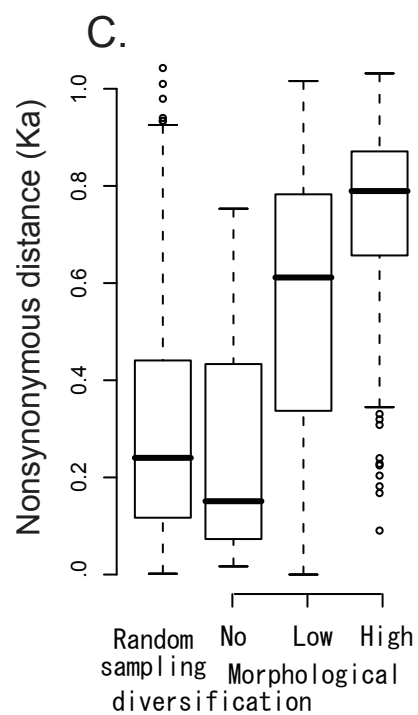

Supplement: Figure S1 — Expression divergence, synonymous, and nonsynonymous distances among random paralogous gene pairs and among paralogous gene pairs with no, low, and high morphological diversification. (A) Relationship between expression divergence and morphological diversification (defined in the main text). Expression divergence is log ((1−R)/(1+R)), where R is the correlation coefficient of paralogous gene pairs among different experimental conditions. (B) Relationship between Ks and morphological diversification. (C) Relationship between Ka and morphological diversification. The random sample included 1000 pairs of paralogs. The distributions of expression divergence, Ks and Ka are shown as box plots with the solid horizontal line indicating the median value, the box representing the inter quartile range (25%–75%), and the dotted line indicating the first to the 99th percentile. (0.25 MB PDF) [file pgen.1000781.s001.pdf]

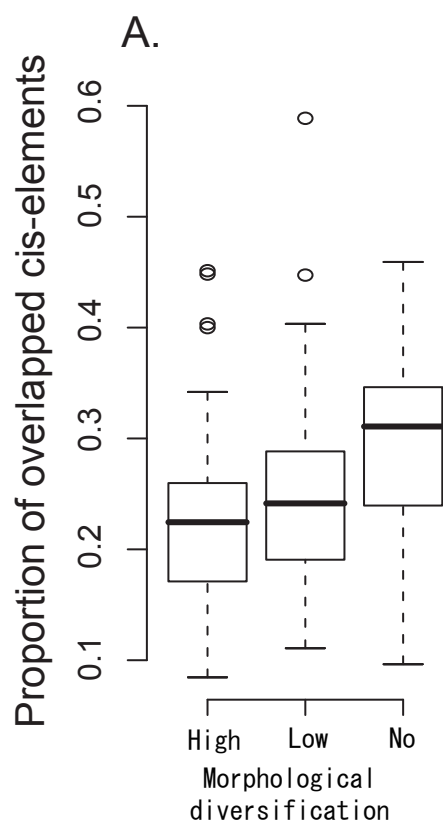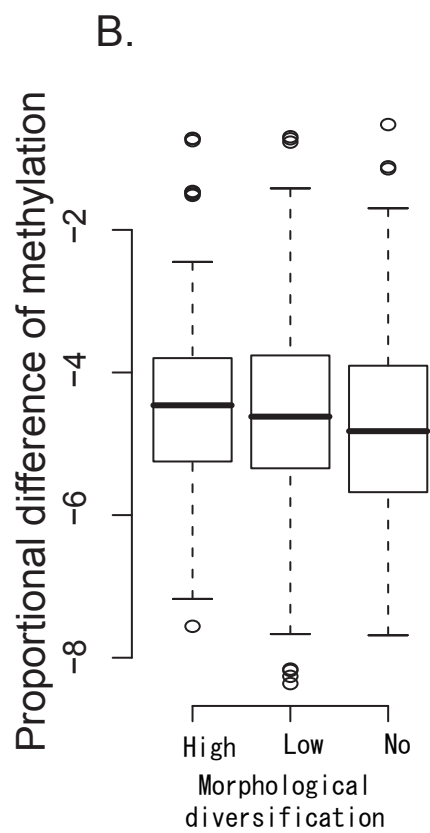

Supplement: Figure S2 — Divergence of cis-regulatory element and methylation of promoter regions among paralogous gene pairs with no, low, and high morphological diversification. (A) Relationship between proportion of overlapped cis-regulatory elements and morphological diversification. The proportion of overlapped cis-regulatory elements is the number of overlapped cis-regulatory elements over the number of observed cis-regulatory elements in promotor regions of two paralogous genes. (B) Relationship between proportional difference of methylation and morphological diversification. The proportional diffrerence of methylation is the difference of proportion of methylated cytosine in promoter regions of two paralogous genes. These distributions are shown as box plots with the solid horizontal line indicating the median value, the box representing the inter quartile range (25%–75%), and the dotted line indicating the first to the 99th percentile. (0.22 MB PDF) [file pgen.1000781.s002.pdf]
