# Supplementary material for: Increased Expression and Protein Divergence in Duplicate Genes Is Associated with Morphological Diversification
Source: PLoS Genet. 2009 Dec 24;5(12):e1000781. doi: 10.1371/journal.pgen.1000781 (PMC2788128; doi:10.1371/journal.pgen.1000781)
Supplement: Table S1 — Paralogous gene pairs with no, low, and high morphological diversification. (0.05 MB PDF) [file pgen.1000781.s003.pdf]

Table S1

Paralogous gene pairs with no morphological diversification

[1]: This gene code is based on TAIR7 (www.arabidopsis.org)

[2]:Types of phenotypic changes (no:no phenotypic effect, S:seed, V:vegitable, R:reproductive)

Paralog A[1]	Paralog B[1]	Phenotype[2] throughout duble knock-out both paralog A and B
AT4G22890	AT4G11960	V
AT5G10490	AT1G58200	V
AT4G32730	AT5G11510	V
AT3G07780	AT5G48160	V
AT2G23150	AT5G67330	V
AT2G16500	AT4G34710	S
AT5G64070	AT5G09350	V
AT3G58780	AT2G42830	R
AT1G75850	AT2G17790	V
AT1G75850	AT3G51310	V
AT2G17790	AT3G51310	V
AT4G25700	AT5G52570	V
AT2G16640	AT3G16620	V
AT2G46340	AT4G11110	V
AT2G46340	AT3G15354	V
AT2G46340	AT1G53090	V
AT4G11110	AT3G15354	V
AT4G11110	AT1G53090	V
AT3G15354	AT1G53090	V
AT1G31340	AT2G35635	S
AT2G37040	AT3G53260	R
AT1G69670	AT1G26830	S
AT1G65660	AT4G37120	S
AT3G19450	AT4G34230	V
AT2G46770	AT3G61910	R
AT1G34210	AT1G71830	R
AT5G06100	AT3G11440	R
AT3G13730	AT4G36380	V
AT1G74560	AT1G18800	V
AT3G49690	AT2G36890	V
AT1G48910	AT1G21430	V
AT1G48910	AT4G32540	V
AT1G48910	AT5G11320	V
AT1G21430	AT4G32540	V
AT1G21430	AT5G11320	V
AT4G32540	AT5G11320	V
AT3G27080	AT5G40930	V
AT3G27080	AT1G27390	V
AT5G40930	AT1G27390	V
AT3G01650	AT5G14420	V
AT1G67230	AT1G13220	V
AT2G17420	AT4G35460	R
AT4G36870	AT2G23760	V
AT5G56280	AT4G26430	V
AT4G19600	AT5G45190	S
AT3G05120	AT3G63010	V
AT3G05120	AT5G27320	V
AT3G63010	AT5G27320	V
AT4G14150	AT3G23670	R
AT4G21690	AT1G15550	R
AT1G03130	AT4G02770	V
AT3G49670	AT5G65700	V
AT3G49670	AT4G20270	V

AT5G65700	AT4G20270	V
AT3G13530	AT3G07980	R
AT5G20830	AT3G43190	V
AT2G22780	AT5G09660	V
AT1G52740	AT3G54560	V
AT5G14070	AT3G02000	R
AT4G21270	AT4G05190	R
AT4G20140	AT5G44700	V
AT1G02880	AT2G44750	V
AT1G23820	AT1G70310	S
AT3G16785	AT3G05630	V
AT3G18030	AT1G48605	S
AT3G22840	AT4G14690	V
AT2G35690	AT4G16760	R
AT3G02230	AT5G15650	R
AT1G58080	AT1G09795	S
AT3G04080	AT5G18280	V
AT3G04080	AT5G18280	V
AT5G18280	AT3G04080	V
AT3G04080	AT5G18280	V
AT5G03300	AT3G09820	S
AT4G26840	AT5G55160	S
AT4G33010	AT2G26080	V
AT2G22310	AT4G39910	R
AT2G25650	AT5G14280	V
AT2G25650	AT4G00270	V
AT5G14280	AT4G00270	V
AT5G49910	AT4G24280	S
AT5G23670	AT3G48780	R
AT5G23670	AT3G48780	R
AT3G48780	AT5G23670	R
AT5G23670	AT3G48780	R
AT4G23650	AT2G17290	V
AT1G27320	AT5G35750	V
AT4G32010	AT2G30470	V
AT2G44900	AT3G60350	V
AT3G50070	AT4G34160	V
AT3G50070	AT5G67260	V
AT4G34160	AT5G67260	V
AT4G00400	AT1G01610	V
AT5G62410	AT3G47460	S

Paralogous gene pairs with low morphological diversification

[1]: This gene code is based on TAIR7 (www.arabidopsis.org)

[2]:Types of phenotypic changes (no:no phenotypic effect, S:seed, V:vegitable, R:reproductive)

Paralog A[1]	Paralog B[1]	Knock-out phenotype[2] of paralog A	Knock-out phenotype[2] of paralog B
AT1G75820	AT1G55610	R	R
AT1G75820	AT5G07280	R	R
AT2G26330	AT2G02220	V	V
AT2G26330	AT5G61480	V	V
AT2G26330	AT3G02130	V	V
AT2G26330	AT4G39400	V	V
AT2G26330	AT4G33430	V	V
AT3G14370	AT1G53700	V	V
AT2G02220	AT2G20300	V	V
AT2G02220	AT5G61480	V	V
AT2G02220	AT4G39400	V	V
AT2G02220	AT4G33430	V	V
AT1G55610	AT5G07280	R	R
AT2G20300	AT4G33430	V	V
AT2G20300	AT1G05700	V	V
AT2G20300	AT1G74490	V	V
AT5G61480	AT4G39400	V	V
AT5G61480	AT4G33430	V	V
AT5G07280	AT1G65380	R	R
AT3G02130	AT4G33430	V	V
AT4G33430	AT1G74490	V	V
AT4G33430	AT2G39660	V	V
AT1G74490	AT2G39660	V	V
AT3G48750	AT2G43790	R	R
AT4G36930	AT5G67110	R	R
AT5G09750	AT4G00120	R	R
AT5G41315	AT1G63650	V	V
AT5G56110	AT5G35550	R	R
AT5G56110	AT4G34990	R	R
AT5G56110	AT1G09540	R	R
AT3G60460	AT5G35550	R	R
AT3G60460	AT1G09540	R	R
AT5G35550	AT4G34990	R	R
AT5G35550	AT1G09540	R	R
AT5G23000	AT4G25560	V	V
AT5G23000	AT5G14750	V	V
AT5G23000	AT5G40330	V	V
AT5G23000	AT3G27920	V	V
AT4G34990	AT1G09540	R	R
AT4G25560	AT5G14750	V	V
AT4G25560	AT5G40330	V	V
AT4G25560	AT3G27920	V	V
AT5G14750	AT5G40330	V	V
AT5G14750	AT5G53200	V	V
AT5G14750	AT3G27920	V	V
AT5G40330	AT2G30420	V	V
AT5G40330	AT3G27920	V	V
AT2G46410	AT4G01060	V	V
AT2G46410	AT5G53200	V	V
AT2G46410	AT2G30420	V	V
AT2G46410	AT2G30432	V	V
AT4G01060	AT5G53200	V	V
AT4G01060	AT2G30420	V	V
AT4G01060	AT2G30432	V	V
AT5G53200	AT2G30420	V	V

AT5G53200	AT2G30432	V	V
AT1G01280	AT5G07990	R	R
AT2G40890	AT4G31500	V	V
AT4G31500	AT1G13710	V	V
AT2G29090	AT5G45340	S	S
AT2G29090	AT4G19230	S	S
AT5G05690	AT3G30180	V	V
AT5G05690	AT4G36380	V	V
AT3G30180	AT4G36380	V	V
AT5G45340	AT4G19230	S	S
AT1G69120	AT1G24260	R	R
AT1G69120	AT5G60910	R	R
AT1G69120	AT3G54340	R	R
AT1G69120	AT4G18960	R	R
AT1G69120	AT5G23260	R	R
AT1G69120	AT5G20240	R	R
AT4G22950	AT2G22540	V	V
AT4G22950	AT4G24540	V	V
AT4G22950	AT3G57230	V	V
AT4G22950	AT5G65050	V	V
AT4G22950	AT5G10140	V	V
AT2G22540	AT4G24540	V	V
AT2G22540	AT3G57230	V	V
AT2G22540	AT5G10140	V	V
AT1G24260	AT5G60910	R	R
AT1G24260	AT4G18960	R	R
AT1G24260	AT5G23260	R	R
AT1G24260	AT5G20240	R	R
AT5G60910	AT3G54340	R	R
AT5G60910	AT4G18960	R	R
AT5G60910	AT5G23260	R	R
AT5G60910	AT5G20240	R	R
AT3G54340	AT4G18960	R	R
AT3G54340	AT5G23260	R	R
AT3G54340	AT5G20240	R	R
AT4G18960	AT5G23260	R	R
AT4G18960	AT5G20240	R	R
AT4G24540	AT3G57230	V	V
AT4G24540	AT5G65050	V	V
AT4G24540	AT5G10140	V	V
AT5G23260	AT5G20240	R	R
AT3G57230	AT5G65050	V	V
AT3G57230	AT5G10140	V	V
AT5G65050	AT5G10140	V	V
AT1G15510	AT4G18750	V	V
AT1G15510	AT5G48910	V	V
AT4G18750	AT5G48910	V	V
AT1G04820	AT1G50010	V	V
AT1G04820	AT4G14960	V	V
AT1G04820	AT5G19770	V	V
AT1G04820	AT5G19780	V	V
AT1G04820	AT1G75780	V	V
AT1G04820	AT5G62700	V	V
AT1G04820	AT5G62690	V	V
AT1G04820	AT5G44340	V	V
AT1G50010	AT4G14960	V	V
AT1G50010	AT5G19770	V	V
AT1G50010	AT5G19780	V	V
AT1G50010	AT1G75780	V	V
AT1G50010	AT5G62700	V	V

AT1G50010	AT5G62690	V	V
AT1G50010	AT5G44340	V	V
AT4G14960	AT5G19770	V	V
AT4G14960	AT5G19780	V	V
AT4G14960	AT1G75780	V	V
AT4G14960	AT5G62700	V	V
AT4G14960	AT5G62690	V	V
AT4G14960	AT5G44340	V	V
AT5G19770	AT1G75780	V	V
AT5G19770	AT5G62700	V	V
AT5G19770	AT5G62690	V	V
AT5G19770	AT5G44340	V	V
AT5G19780	AT1G75780	V	V
AT5G19780	AT5G62700	V	V
AT5G19780	AT5G62690	V	V
AT5G19780	AT5G44340	V	V
AT1G75780	AT5G62700	V	V
AT1G75780	AT5G62690	V	V
AT1G75780	AT5G44340	V	V
AT5G62700	AT5G44340	V	V
AT5G62690	AT5G44340	V	V
AT4G32410	AT3G03050	V	V
AT4G32410	AT5G64740	V	V
AT4G32410	AT5G05170	V	V
AT4G32410	AT1G02730	V	V
AT4G32410	AT4G18780	V	V
AT4G32410	AT5G17420	V	V
AT4G32410	AT4G39350	V	V
AT3G03050	AT5G64740	V	V
AT3G03050	AT5G05170	V	V
AT3G03050	AT1G02730	V	V
AT3G03050	AT4G18780	V	V
AT3G03050	AT5G17420	V	V
AT3G03050	AT4G39350	V	V
AT5G64740	AT5G05170	V	V
AT5G64740	AT1G02730	V	V
AT5G64740	AT4G18780	V	V
AT5G64740	AT5G17420	V	V
AT5G64740	AT4G39350	V	V
AT5G05170	AT1G02730	V	V
AT5G05170	AT4G18780	V	V
AT5G05170	AT5G17420	V	V
AT5G05170	AT4G39350	V	V
AT1G02730	AT4G18780	V	V
AT1G02730	AT5G17420	V	V
AT1G02730	AT4G39350	V	V
AT4G18780	AT5G17420	V	V
AT4G18780	AT4G39350	V	V
AT5G17420	AT4G39350	V	V
AT5G64580	AT2G30950	S	S
AT5G53170	AT5G42270	V	V
AT4G29040	AT5G42270	V	V
AT5G07290	AT5G61960	V	V
AT5G51810	AT4G25420	V	V
AT5G51810	AT1G15550	V	V
AT1G12980	AT1G24590	S	S
AT4G37750	AT4G36920	R	R
AT5G53950	AT3G15170	S	S
AT5G53950	AT1G76420	S	S
AT3G15170	AT1G76420	S	S

AT2G27300	AT1G69490	V	V
AT1G04250	AT3G23050	V	V
AT1G04250	AT4G14560	V	V
AT3G23050	AT1G04240	V	V
AT3G23050	AT4G14560	V	V
AT1G04240	AT4G14560	V	V
AT3G51570	AT5G45250	V	V
AT3G51570	AT5G17880	V	V
AT3G51570	AT5G44870	V	V
AT5G45250	AT5G17880	V	V
AT5G45250	AT5G44870	V	V
AT5G17880	AT5G44870	V	V
AT3G27000	AT3G18780	V	V
AT3G27000	AT1G13180	V	V
AT3G18780	AT1G13180	V	V
AT2G41940	AT3G58070	V	V
AT2G41940	AT5G06650	V	V
AT3G58070	AT5G06650	V	V
AT5G01410	AT2G38230	V	V
AT5G04130	AT5G04110	V	V
AT5G04130	AT3G10270	V	V
AT5G04110	AT3G10270	V	V
AT1G02280	AT5G05000	V	V
AT1G41830	AT4G12420	V	V
AT1G71230	AT1G22920	V	V
AT1G05470	AT1G34120	V	V
AT1G05470	AT5G65090	V	V
AT1G34120	AT5G65090	V	V
AT1G80010	AT1G52520	V	V
AT5G10510	AT1G51190	V	V
AT3G10140	AT2G19490	V	V
AT5G06170	AT1G22710	V	V
AT2G26690	AT1G32450	V	V
AT2G26690	AT1G12110	V	V
AT1G32450	AT1G12110	V	V
AT2G45190	AT1G23420	R	R
AT5G20850	AT3G22880	R	R
AT3G22880	AT2G45280	R	R
AT1G64760	AT5G58090	R	R
AT1G61720	AT5G42800	R	R
AT1G28380	AT1G29690	V	V
AT4G17730	AT5G46860	V	V
AT1G65480	AT4G20370	V	V
AT1G68050	AT5G57360	V	V
AT1G09570	AT5G35840	V	V
AT1G05580	AT2G31910	V	V
AT1G68530	AT1G01120	V	V
AT1G54490	AT1G75660	V	V
AT5G01040	AT2G29130	V	V
AT4G00730	AT4G04890	V	V
AT5G62500	AT3G47690	V	V
AT2G42890	AT1G29400	V	V
AT1G65580	AT1G05630	V	V
AT1G01420	AT4G01070	V	V
AT4G18370	AT5G39830	V	V
AT1G17840	AT1G51500	V	V
AT5G57050	AT4G26080	V	V
AT5G66570	AT3G50820	V	V
AT5G16050	AT2G42590	V	V
AT1G15820	AT1G19150	V	V

Paralogous gene pairs with high morphological diversification

[1]: This gene code is based on TAIR7 (www.arabidopsis.org)

[2]:Types of phenotypic changes (no:no phenotypic effect, S:seed, V:vegitable, R:reproductive)

Paralog A[1]	Paralog B[1]	Knock-out phenotype [2] of paralog A	Knock-out phenotype [2] of paralog B
AT1G75820	AT2G26330	R	V
AT1G75820	AT2G02220	R	V
AT1G75820	AT3G19700	R	S
AT1G75820	AT5G61480	R	V
AT1G75820	AT4G39400	R	V
AT1G75820	AT4G33430	R	V
AT2G26330	AT1G55610	V	R
AT2G26330	AT3G19700	V	S
AT2G26330	AT5G07280	V	R
AT3G14370	AT2G34650	V	S
AT1G63700	AT4G08500	S	V
AT2G02220	AT1G55610	V	R
AT2G02220	AT5G07280	V	R
AT1G55610	AT3G19700	R	S
AT1G55610	AT5G61480	R	V
AT1G55610	AT3G02130	R	V
AT1G55610	AT4G39400	R	V
AT3G19700	AT5G61480	S	V
AT3G19700	AT5G07280	S	R
AT3G19700	AT4G33430	S	V
AT4G05200	AT1G74490	R	V
AT4G05200	AT2G39660	R	V
AT5G61480	AT5G07280	V	R
AT5G07280	AT3G02130	R	V
AT5G07280	AT4G39400	R	V
AT4G33430	AT3G51550	V	R
AT5G54380	AT3G51550	V	R
AT3G48750	AT5G63610	R	V
AT3G48750	AT4G18710	R	V
AT2G43790	AT5G63610	R	V
AT2G43790	AT4G18710	R	V
AT1G53700	AT2G34650	V	S
AT4G36930	AT1G09530	R	V
AT5G41315	AT4G09820	V	R
AT2G46970	AT5G67110	V	R
AT5G67110	AT5G61270	R	V
AT1G63650	AT4G09820	V	R
AT5G56110	AT5G23000	R	V
AT5G56110	AT4G25560	R	V
AT5G56110	AT5G14750	R	V
AT5G56110	AT5G40330	R	V
AT5G56110	AT3G27920	R	V
AT3G60460	AT4G25560	R	V
AT3G60460	AT5G14750	R	V
AT3G60460	AT5G40330	R	V
AT3G60460	AT3G27920	R	V
AT5G35550	AT5G23000	R	V
AT5G35550	AT4G25560	R	V
AT5G35550	AT5G14750	R	V
AT5G35550	AT5G40330	R	V
AT5G35550	AT3G27920	R	V
AT5G23000	AT4G34990	V	R
AT5G23000	AT1G09540	V	R
AT4G34990	AT4G25560	R	V

AT4G34990	AT5G14750	R	V
AT4G34990	AT5G40330	R	V
AT4G34990	AT3G27920	R	V
AT4G25560	AT1G09540	V	R
AT5G14750	AT1G09540	V	R
AT1G09540	AT5G40330	R	V
AT1G09540	AT3G27920	R	V
AT4G36260	AT3G51060	V	R
AT1G01280	AT2G40890	R	V
AT5G07990	AT2G40890	R	V
AT5G07990	AT4G31500	R	V
AT5G07990	AT1G13710	R	V
AT2G29090	AT5G05690	S	V
AT2G29090	AT4G36380	S	V
AT5G05690	AT5G45340	V	S
AT5G05690	AT4G19230	V	S
AT3G30180	AT5G45340	V	S
AT3G30180	AT4G19230	V	S
AT5G45340	AT4G36380	S	V
AT4G36380	AT4G19230	V	S
AT1G69120	AT4G22950	R	V
AT1G69120	AT2G22540	R	V
AT1G69120	AT4G24540	R	V
AT1G69120	AT3G57230	R	V
AT1G69120	AT5G65050	R	V
AT1G69120	AT5G10140	R	V
AT4G22950	AT1G24260	V	R
AT4G22950	AT5G60910	V	R
AT4G22950	AT4G18960	V	R
AT4G22950	AT5G23260	V	R
AT2G22540	AT1G24260	V	R
AT2G22540	AT3G54340	V	R
AT2G22540	AT4G18960	V	R
AT2G22540	AT5G23260	V	R
AT2G22540	AT5G20240	V	R
AT1G24260	AT4G24540	R	V
AT1G24260	AT3G57230	R	V
AT1G24260	AT5G65050	R	V
AT1G24260	AT5G10140	R	V
AT5G60910	AT4G24540	R	V
AT5G60910	AT3G57230	R	V
AT5G60910	AT5G65050	R	V
AT5G60910	AT5G10140	R	V
AT3G54340	AT4G24540	R	V
AT3G54340	AT5G10140	R	V
AT4G18960	AT4G24540	R	V
AT4G18960	AT3G57230	R	V
AT4G18960	AT5G65050	R	V
AT4G18960	AT5G10140	R	V
AT4G24540	AT5G23260	V	R
AT4G24540	AT5G20240	V	R
AT5G23260	AT3G57230	R	V
AT5G23260	AT5G65050	R	V
AT5G23260	AT5G10140	R	V
AT3G57230	AT5G20240	V	R
AT5G20240	AT5G10140	R	V
AT1G08070	AT1G15510	S	V
AT1G08070	AT4G18750	S	V
AT1G08070	AT5G48910	S	V

AT1G08070	AT5G40410	S	R
AT1G15510	AT5G40410	V	R
AT4G18750	AT5G40410	V	R
AT5G48910	AT5G40410	V	R
AT4G32410	AT4G38190	V	S
AT3G03050	AT4G38190	V	S
AT5G64740	AT4G38190	V	S
AT5G05170	AT4G38190	V	S
AT1G02730	AT4G38190	V	S
AT4G38190	AT4G18780	S	V
AT4G38190	AT5G17420	S	V
AT4G38190	AT4G39350	S	V
AT5G64580	AT5G42270	S	V
AT5G53170	AT2G30950	V	S
AT2G30950	AT5G42270	S	V
AT5G51810	AT3G51240	V	R
AT4G25420	AT3G51240	V	R
AT1G15550	AT3G51240	V	R
AT1G15750	AT3G16830	S	V
AT5G53950	AT1G69490	S	V
AT3G15170	AT2G27300	S	V
AT3G15170	AT1G69490	S	V
AT1G76420	AT1G69490	S	V
AT1G04250	AT1G04550	V	S
AT3G23050	AT1G04550	V	S
AT1G04240	AT1G04550	V	S
AT4G14560	AT1G04550	V	S
AT3G60830	AT3G18780	S	V
AT3G27000	AT1G18450	V	R
AT1G18450	AT3G18780	R	V
AT1G18450	AT1G13180	R	V
AT2G41940	AT5G14010	V	R
AT5G14010	AT3G58070	R	V
AT1G19850	AT5G20730	S	V
AT1G19850	AT5G37020	S	R
AT5G20730	AT5G37020	V	R
AT5G06170	AT1G71890	V	S
AT1G71890	AT1G22710	S	V
AT1G61720	AT1G15950	R	V
AT5G42800	AT1G15950	R	V
AT4G17730	AT5G16830	V	R
AT5G46860	AT5G16830	V	R
AT1G65480	AT5G03840	V	R
AT4G20370	AT5G03840	V	R
AT5G57090	AT1G73590	V	R
AT3G06860	AT4G29010	V	R
AT2G40220	AT3G60490	S	V
AT2G01420	AT1G70940	S	V
AT1G17260	AT3G47950	R	V
AT4G00220	AT1G65620	S	V
