# Supplementary material for: Increased Expression and Protein Divergence in Duplicate Genes Is Associated with Morphological Diversification
Source: PLoS Genet. 2009 Dec 24;5(12):e1000781. doi: 10.1371/journal.pgen.1000781 (PMC2788128; doi:10.1371/journal.pgen.1000781)
Supplement: Table S2 — Statistical difference (P. values) in Figure 2, Figure S1, and Figure S2. (0.01 MB PDF) [file pgen.1000781.s004.pdf]

Table S2

Statistical difference (P. values) between every pair in random paralogous group and no, low and high morphological diversification groups in the wilcoxon test.

In Figure S1-A

	No	Low	High	Random
No		3.1×10^{-4} *	2.2×10^{-16} *	2.7×10^{-4} *
Low			7.2×10^{-9} *	0.20
High				2.2×10^{-16} *
Random				

* Significant difference at P=0.01.

In Figure S1-B

	No	Low	High	Random
No		3.0×10^{-16} *	3.5×10^{-25} *	0.49
Low			1.1×10^{-5} *	1.7×10^{-37} *
High				7.0×10^{-49} *
Random				

* Significant difference at P=0.01.

In Figure S1-C

	No	Low	High	Random
No		3.0×10^{-18} *	3.9×10^{-33} *	5.6×10^{-3} *
Low			2.8×10^{-13} *	3.8×10^{-34} *
High				3.3×10^{-63} *
Random				

* Significant difference at P=0.01.

In Figure 2-A

	No	Low	High	Random
No		4.7×10^{-7} *	1.5×10^{-16} *	7.0×10^{-4} *
Low			9.7×10^{-9} *	4.8×10^{-4} *
High				2.4×10^{-22} *
Random				

* Significant difference at P=0.01.

In Figure 2-B

	No	Low	High	Random
No		1.2×10^{-9}*	2.7×10^{-19} *	5.4×10^{-7} *
Low			3.9×10^{-5} *	3.0×10^{-3} *
High				1.1×10^{-13} *
Random				

* Significant difference at P=0.01.

In Figure 2-C

	No	Low	High	Random
No		0.06	0.09	0.11
Low			0.87	0.61
High				0.83
Random				

* Significant difference at P=0.01.

In Figure S2-A

	No	Low	High
No		3.3×10^{-6} *	3.3×10^{-9} *
Low			0.02 *

* Significant difference at P=0.05.

In Figure S2-B

	No	Low	High
No		0.34	0.20
Low			0.62

* Significant difference at P=0.05.
