# Supplementary material for: Increased Expression and Protein Divergence in Duplicate Genes Is Associated with Morphological Diversification
Source: PLoS Genet. 2009 Dec 24;5(12):e1000781. doi: 10.1371/journal.pgen.1000781 (PMC2788128; doi:10.1371/journal.pgen.1000781)
Supplement: Table S3 — Number of paralogous gene pairs with a high divergence rate of protein sequence (more than the top 10% of Ed of paralogous gene pairs without morphological diversification) and/or expression (more than the top 10% of Ka/Ks ratios of paralogous gene pairs without morphological diversification) in the high and low morphological diversification groups. (0.03 MB PDF) [file pgen.1000781.s005.pdf]

Table S3

Morphological diversification	Protein	Expression		P-values[c]
		Divergent	Not divergent	
High	Divergent	44	35 (55%)[b]	0.45
	Not divergent	29 (45%)[a]	35	
Low	Divergent	17	50 (64%)[b]	0.004
	Not divergent	28 (36%)[a]	82	
Either high or low	Divergent	61	85 (63%)[b]	0.02
	Not divergent	57 (37%)[a]	117	

[a] Proportion of paralogous gene pairs with a higher expression divergence but no protein divergence

[b] Proportion of paralogous gene pairs with a higher protein divergence but no expression divergence

[c] Null hypothesis is that proportion of paralogous gene pairs with a higher expression divergence is the same proportion of paralogous gene pairs with a higher protein divergence.
